# PIM kinases inhibit AMPK activation and promote tumorigenicity by phosphorylating LKB1

**DOI:** 10.1186/s12964-021-00749-4

**Published:** 2021-06-30

**Authors:** Kwan Long Mung, William B. Eccleshall, Niina M. Santio, Adolfo Rivero-Müller, Päivi J. Koskinen

**Affiliations:** 1 Department of Biology, University of Turku, Vesilinnantie 5, 20500 Turku, Finland; 2 Faculty of Science and Engineering/Cell Biology, Åbo Akademi University, Turku, Finland; 3 Present Address: Department of Biochemistry and Molecular Biology, Medical University of Lublin, Lublin, Poland

**Keywords:** PIM kinases, LKB1, AMPK, Phosphorylation, Prostate cancer, Breast cancer, CAM model

## Abstract

**Background:**

The oncogenic PIM kinases and the tumor-suppressive LKB1 kinase have both been implicated in the regulation of cell growth and metabolism, albeit in opposite directions. Here we investigated whether these kinases interact with each other to influence AMPK activation and tumorigenic growth of prostate and breast cancer cells.

**Methods:**

We first determined how PIM and LKB1 kinases affect AMPK phosphorylation levels. We then used in vitro kinase assays to demonstrate that LKB1 is phosphorylated by PIM kinases, and site-directed mutagenesis to identify the PIM target sites in LKB1. The cellular functions of PIM and LKB1 kinases were evaluated using either pan-PIM inhibitors or CRISPR/Cas9 genomic editing, with which all three PIM family members and/or LKB1 were knocked out from PC3 prostate and MCF7 breast cancer cell lines. In addition to cell proliferation assays, we examined the effects of PIM and/or LKB1 loss on tumor growth using the chick embryo chorioallantoic membrane (CAM) xenograft model.

**Results:**

We provide both genetic and pharmacological evidence to demonstrate that inhibition of PIM expression or activity increases phosphorylation of AMPK at Thr172 in both PC3 and MCF7 cells, but not in their derivatives lacking LKB1. This is explained by our observation that all three PIM family kinases can phosphorylate LKB1 at Ser334. Wild-type LKB1, but not its phosphodeficient derivative, can restore PIM inhibitor-induced AMPK phosphorylation in LKB1 knock-out cells. In the CAM model, loss of LKB1 enhances tumorigenicity of PC3 xenografts, while cells lacking both LKB1 and PIMs exhibit slower proliferation rates and form smaller tumors.

**Conclusion:**

PIM kinases are novel negative regulators of LKB1 that affect AMPK activity in an LKB1-dependent fashion. The impairment of cell proliferation and tumor growth in cells lacking both LKB1 and PIMs indicates that these kinases possess a shared signaling role in the context of cancer. These data also suggest that PIM inhibitors may be a rational therapeutic option for LKB1-deficient tumors.
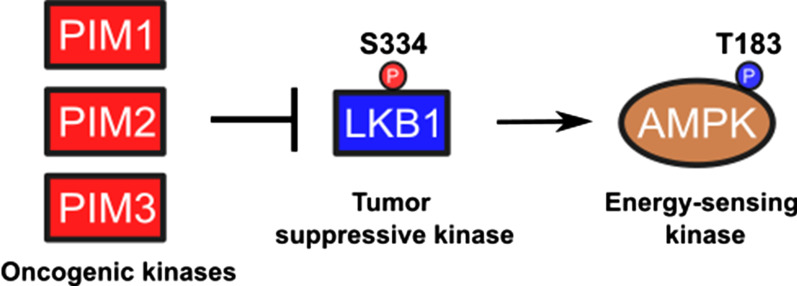

**Video Abstract**

**Supplementary Information:**

The online version contains supplementary material available at 10.1186/s12964-021-00749-4.

## Background

The three serine/threonine-specific PIM family members (PIM1, PIM2, and PIM3) are highly homologous and in part functionally redundant [1–3]. As PIM kinases are constitutively active in cells [4], their catalytic activities correlate well with their protein expression levels. These oncogenic kinases are often overexpressed in solid tumors or haematological malignancies, in which they promote cell proliferation, survival, motility and metabolism via phosphorylation-dependent activation or inactivation of a wide variety of substrates, such as the NFATC1 and NOTCH1 transcriptional regulators, the CDKN1A and CDKN1B cell cycle inhibitors, the BAD pro-apoptotic protein, the CXCR4 chemokine receptor, the CAPZ actin capping proteins, and the AKT1S1 and EIF4EBP1 translational inhibitors [1–3, 5]. Accordingly, PIM kinases have emerged as attractive targets for cancer therapy, especially as they possess a structurally unique ATP-binding pocket [4], and as PIM triple knock-out (TKO) mice are viable and fertile with only a mild reduction in body size [6]. This phenotype may at least partially be due to reduced cytokine responses [7] and a diminished glycolytic phenotype [8].

The serine/threonine-specific liver kinase B1 (LKB1), encoded by the *STK11* gene, is a tumor suppressor, which is mutated in patients with the hereditary Peutz-Jeghers syndrome [9, 10]. Somatic inactivating mutations have also been found in sporadic tumors: 5–17% of non-small cell lung carcinomas [11–13], 5% of pancreatic cancers and melanomas [14–16] and around 20% of cervical cancers [17, 18]. Furthermore, *STK11* has been identified as the third most frequently mutated gene in human lung adenocarcinoma, following *TP53* and *KRAS* [19]. By contrast, LKB1 mutations have rarely been reported from breast, colorectal or gastric cancer [9]. The tumor suppressor function of LKB1 is largely attributed to its ability to phosphorylate the AMP-activated protein kinase (AMPK) [20–22] and 12 other closely related kinases [23]. AMPK in turn is a heterotrimeric protein comprising of a catalytic α subunit and regulatory β and γ subunits [24]. In response to changes in the AMP/ATP ratio resulting e.g. from energy deprivation, LKB1 phosphorylates the α subunit of AMPK at a conserved threonine site (commonly stated as Thr172 because of its pivotal finding in rats [25], while the corresponding site in the human protein is Thr183). Phosphorylation of AMPK increases its catalytic activity more than 100-fold in vitro [26], and in cells this allows it to inhibit anabolic biosynthetic pathways and to promote catabolic processes to restore the energy balance in favour of ATP production [24, 27]. Remarkably, failure to activate AMPK in response to energy stress has been proposed as an explanation for the massive cell death that occurs in LKB1-deficient tumors after treatment with metabolic inhibitors, such as metformin or phenformin [28, 29]. Interestingly, inhibition of PIM expression or activity has been shown to increase AMPK phosphorylation, possibly via LKB1 [30], but the exact mechanism behind this phenomenon has remained unclear.

As cancer cell growth and metabolism are regulated by the balance between oncogenic (e.g. PIM) and tumor-suppressive (e.g. LKB1) kinases, both overexpression of PIM kinases and loss of LKB1 expression are expected to promote tumorigenesis. In the present study with prostate and breast cancer cell lines expressing PIM and LKB1 kinases, we demonstrate that PIM kinases act as upstream kinases of LKB1 and that Ser334 in LKB1 is their phosphorylation target site. Both pharmacological and CRISPR/Cas9-based approaches reveal that inhibition of expression or activity of all three PIM family members upregulates AMPK activity in an LKB1-dependent manner. Finally, double knock-out of both LKB1 and PIM kinases led to a striking reduction in cell proliferation and tumor growth, raising possibilities for PIM-targeted pharmaceutical interventions in suppressing the growth of LKB1-deficient tumors.

## Methods

### Cell culture, reagents and DNA constructs

MCF7 breast cancer, HeLa cervical cancer and PC3 prostate cancer cells were obtained from American Type Culture Collection (Manassas, VA). Construction and maintenance of FDCP1-derived myeloid cell lines have been described previously [31]. MCF7 and HeLa cells were cultured in Dulbecco’s modified Eagle’s medium (DMEM), and PC3 and FDCP1 cells in RPMI-1640 medium (Sigma-Aldrich, St. Louis, MI, USA). Both media were supplemented with L-glutamine, 10% fetal bovine serum and antibiotics. MEM Non-Essential Amino Acids (Gibco, #11140050; Thermo Fisher Scientific, Waltham, MA, USA) and sodium pyruvate (Gibco, #11360070; Thermo Fisher Scientific) were further added to RPMI-1640 medium to facilitate cell growth. To study effects of nutrient deprivation, glucose-free medium was supplemented with different concentrations of glucose. FuGENE® HD Transfection Reagent (Promega, Madison, WI, USA) was used for plasmid transfection according to the manufacturer’s protocol. PIM-selective small molecule inhibitors DHPCC9 [32, 33] and AZD1208 (AstraZeneca, Cambridge, UK) were diluted in DMSO. Expression vectors pcDNA™3.1/V5-His-C, pGEX-6P-1 and pTagRFP-N for wild-type (WT) human PIM kinases have been described previously [34]. Expression vectors pcDNA™6.2/N-EmGFP-DEST-LKB1 and pDEST™15-LKB1 were acquired from the Genome Biology Unit core facility (HiLIFE Helsinki Institute of Life Science, Helsinki, Finland). His-tagged LKB1 construct was prepared by subcloning the LKB1 coding region from pcDNA™6.2/N-EmGFP-DEST-LKB1 to pRFSDuet-1 vector. Site-directed mutagenesis of LKB1 was performed by Ultra Pfu DNA Polymerase (Stratagene, San Diego, CA, USA) according to the manufacturer’s protocol. The primers used are described in Supplementary material (Additional file 1: Table S1).

### Establishment of stable knock-out cell lines

The CRISPR-Cas9 genome editing technique [35] was used to create stable knock-out cell lines. CRISPOR (http://crispor.tefor.net/) online software was used to design single guide RNA (sgRNA) sequences. These sequences were acquired as gBlocks® gene fragments (Integrated DNA Technologies, Coralville, Iowa, USA) and ligated into the BbsI-digested pSpCas9(BB)-2A-Puro (PX459) vector or pSpCas9(BB)-2A-GFP (PX458) to simultaneously express two sgRNAs. Transfected cells were either selected for 3–7 days with puromycin or by single cell sorting of GFP-positive cells into 96-well plates with the FACSAria cell sorter (Becton Dickinson, Franklin Lakes, NJ, USA). Knock-out cell screening was done by PCR amplification of the genomic DNA regions surrounding the CRISPR/Cas9 target sites. Genomic DNA extraction and PCR amplification were performed by using Mouse Direct PCR Kit (B40013; Bio-Connect, TE Huissen, The Netherlands) according to the manufacturer´s protocol, with PCR annealing temperature set to 60 °C and extension time to 1 min. The sequencing strategies and gel electrophoresis results are presented in Supplementary material (Additional file 2: Figure S1 and S2), as are also the sgRNA sequences and sequencing primers (Additional file 1: Table S2 and S3). After knocking out individual PIM family members, triple PIM kinase knock-out (TKO) cell lines were generated by sequentially knocking out additional genes.

### Expression of GST-tagged or His-tagged fusion proteins in *Escherichia coli*

pDEST™15 plasmids (expressing GST-LKB1), pGEX-6P-1 plasmids (expressing GST-PIMs) and pRFSDuet-1 plasmid (expressing His-LKB1) were transformed into BL21 *E. coli* strain for protein production. Overnight bacterial cultures were grown at 30 °C until OD_600_ of 0.6. Isopropyl-β-d-galactosidase (250 µM; Sigma-Aldrich) was added to induce protein expression, and the cells were cultured for another 4 h (GST-PIMs) or 24 h (GST-LKB1, His-LKB1). The follow-up purification steps of GST-tagged and His-tagged protein have been described previously [5].

### Western blotting

Cells were lysed for 10 min in ice-cold 50 mM Tris–HCl, pH 8.0 buffer containing 150 mM NaCl, 2 mM EDTA, 1% NP-40, 5 mM NaF, 1 mM Na_3_VO_4_, 1 mM PMSF and Mini EDTA-free protease inhibitor tablet (Roche, Basel, Switzerland). Supernatants were collected after 10 s centrifugation at 21,000 × g. Protein concentrations were determined using the Bio-Rad Protein Assay Dye Reagent or Pierce™ BCA Protein Assay Kit according to manufacturers’ protocols. Protein aliquots (20-60 μg) were separated by 10% SDS-PAGE and transferred onto a PVDF membrane (Millipore, Burlington, MA, USA). The membranes were incubated overnight at + 4 °C with primary antibodies (Additional file 1: Table S4). Secondary antibody staining (1:5000) was performed for 1 h at RT with HRP-linked goat anti-mouse IgG #7076 or goat anti-rabbit IgG #7074 antibodies (Cell Signaling Technology, Beverly, MA, USA). For immunoprecipitation of Flag-tagged proteins, 0.2–1 mg of protein lysate was incubated with 10 μl of anti-Flag® M2 affinity agarose gel (#A2220, Sigma-Aldrich). After 1 h rotation at + 4 °C, the agarose gel was washed three times with the lysis buffer. Samples were prepared for Western blotting by adding of 2 × Laemmli Sample Buffer directly to the agarose gel and by heating the samples for 10 min at + 95 °C prior to gel loading. Chemiluminescence was detected by Bio-Rad Clarity or Clarity Max ECL Western Blotting Substrates. Results were visualised with the ChemiDoc™ MP Imaging System and analysed with Image Lab software Version 5.2.1 (Bio-Rad Laboratories, Inc., Hercules, CA, USA).

### Nuclear/cytoplasmic fractionation

Nearly confluent cells (~ 80% confluence) were collected from 10 cm plates by scraping them into 1 ml aliquots of PBS. After 10 s centrifugation at 21,000 × g, supernatants were discarded and the pellets were lysed for 15 min in 500 μl of lysis buffer: 10 mM Tris–HCl, pH 7.5, 10 mM NaCl, 3 mM MgCl_2_, 0.5% Nonidet P-40, 5 mM NaF, 1 mM PMSF and mini EDTA-free protease inhibitor tablet. After centrifugation at 500 × g for 5 min at + 4 °C, the supernatants contained the cytoplasmic compartments, while the nuclei were in the pellets. The pellets were washed three times with 500 μl lysis buffer and centrifuged each time at 500 × g for 5 min at + 4 °C, after which they were suspended in 200 μl of lysis buffer and sonicated for 30 s. After an additional centrifugation at 500 × g for 1 min, the supernatants were collected which contained nuclear fractions. The cytoplasm-containing solutions were centrifuged at 12,000 × g for 15 min at + 4 °C, after which the supernatant was collected. Laminin A/C and beta-tubulin were used as nuclear and cytosolic markers, respectively, to evaluate fractionation efficiency.

### In vitro kinase assays

The procedure for performing radioactive in vitro kinase assays has been described previously [36]. Briefly, 0.5–2.0 μg of PIM kinase and its substrate were used in each reaction. Samples were separated by SDS-PAGE and stained by Page Blue™ protein staining solution (#24620, Thermo Fisher Scientific). Additional in vitro kinase assays were performed similarly, but without radioactivity. Band intensities were quantitated by the Image Lab software Version 5.2.1 (Bio-Rad).

### Fluorescence-lifetime imaging microscopy (FLIM)

FLIM was carried out as previously described [34] to probe for intracellular protein–protein interactions. Briefly, cells were plated on coverslips and transiently transfected with RFP- or GFP-tagged expression vectors. After 24 h, samples were fixed with 4% paraformaldehyde, washed with PBS and mounted with Mowiol. Samples were imaged by using the Lambert Instruments LIFA FLIM system with the Carl Zeiss AxioImager microscope and LI-FLIM software (Lambert Instruments BV, Groningen, The Netherlands). All imaging was performed at room temperature.

### Proximity ligation assay (PLA)

Cell samples seeded on coverslips were fixed for 10 min with 4% paraformaldehyde, washed twice with PBS, permeabilised with 0.1% Triton X-100 in PBS for 10 min and then washed twice with PBS. Thereafter, the assays were continued using the Duolink® In Situ Detection Reagent kit (DUO9207, Sigma-Aldrich) according to manufacturer’s instructions. Samples were imaged by the Nikon fluorescent microscope with NIS-Elements AR software (Nikon, Tokyo, Japan) and analysed by ImageJ/Fiji.

### Chick chorioallantoic membrane (CAM) model

The chick embryo chorioallantoic membrane (CAM) model [37] was used for in vivo study of tumor development. PC3 and MCF7 cells (0.5–2 × 10^6^) were trypsinised from cell plates, washed with ice-cold PBS twice and mixed 1:1 with Matrigel (356,231; Corning™, NY, USA). A 20 μl aliquot of the solution was added onto each CAM of a fertilized chicken egg on embryonal development day 8 (EDD8). On EDD14, the tumors were excised and weighed immediately.

### IncuCyte analysis

Cells were seeded in a 96-well plate at a density of 3500 cells per well. After an overnight incubation, they were treated with DMSO or DHPCC-9 and imaged every 2 h using the IncuCyte S3 Live-Cell Analysis System (Essen BioScience, Ltd., Newark, United Kingdom). Phase images were acquired and the percentage of confluence of the cell layers was analysed using the IncuCyte® Software (v2019B) Basic Analyzer module.

### In silico analysis

The PhosphoSitePlus® database (https://phosphosite.org, Cell Signaling Technology, Inc., Danvers, MA, USA) was used to search for potential phosphorylation sites. IST Online™ (https://ist.medisapiens.com/) was used to generate gene expression data derived from patient samples.

### Statistical analysis and figure preparation

Bar graphs or scatter plots were produced by GraphPad Prism 6.0 and results were analysed by Student’s t-test. Significant differences (*p* < 0.05 and *p* < 0.01) were marked by * and **, respectively. Error bars represent standard deviations. Inkscape was used for figure preparation.

## Results

### PIM inhibition increases LKB1-dependent phosphorylation of AMPK

**Fig. 1:**
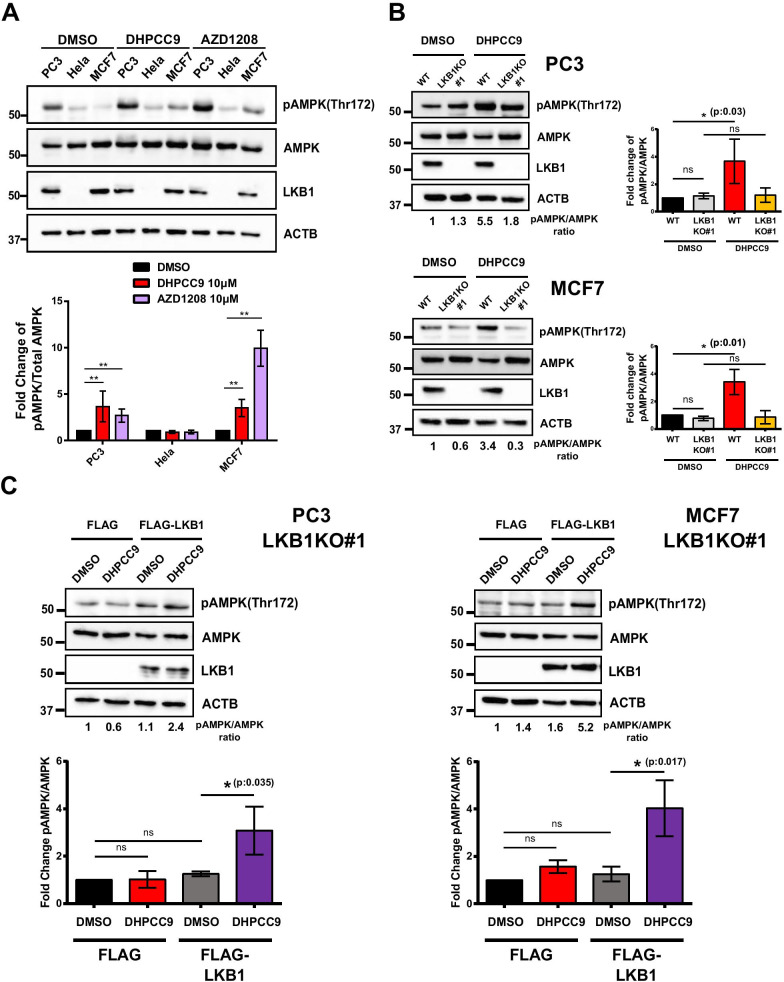
AMPK phosphorylation is enhanced by PIM inhibitors in an LKB1-dependent fashion. **a** PC3, Hela and MCF7 cells were treated for 24 h with DMSO (0.1%) or either DHPCC9 or AZD1208 pan-PIM inhibitor (10 μM in 0.1% DMSO) and subjected to Western blotting with antibodies against phospho-AMPK (Thr172), AMPK or LKB1. ACTB staining was used as a loading control. Shown in the graph are representative images together with graphs, where the relative levels of phosphorylated versus total AMPK were quantitated in comparison to DMSO-treated control samples (average values ± SD, n = 3). **b** Wild-type (WT) PC3 or MCF7 cells or their knock-out derivatives lacking LKB1 (LKB1KO) were treated for 24 h with DMSO or 10 μM DHPCC9, and subjected to Western blotting. Shown in the graphs are relative AMPK phosphorylation levels in comparison to DMSO-treated WT samples (average values ± SD, n = 3). **c** LKB1KO derivatives of PC3 or MCF7 cells were transiently transfected with FLAG or FLAG-LKB1 plasmids, treated for 24 h with DMSO or 10 μM DHPCC9, and subjected to Western blotting. Shown in the graphs are relative AMPK phosphorylation levels in comparison to DMSO-treated FLAG-transfected samples (average values ± SD, n = 3)

To investigate in more detail whether PIM kinases negatively regulate AMPK phosphorylation and activation, we used a pharmacological approach to inhibit PIM activity in PC3 prostate cancer, HeLa cervical cancer and MCF7 breast cancer cell lines. Cells were treated with either DMSO or 10 µM concentrations of two structurally distinct small molecule pan-PIM inhibitors, DHPCC9 or AZD1208, which inhibit the catalytic activity of all three PIM family members [33, 38]. The relative phosphorylation level of AMPK was determined 24 h later by Western blotting with antibodies against AMPK or its phosphorylated Thr172 residue. As shown in Fig. 1a, AMPK was expressed at a similar level in all three cell lines, but it was more prominently phosphorylated in PC3 cells than in the others. When PIM activity was inhibited by either DHPCC9 or AZD1208, AMPK phosphorylation was significantly enhanced in both PC3 and MCF7 cells, but not in HeLa cells. These results could be explained by the observed expression of the AMPK upstream kinase LKB1 in PC3 and MCF7 cells, but not in HeLa cells. Indeed, restoration of LKB1 expression in HeLa cells resulted in increased AMPK phosphorylation in response to treatment with DHPCC9 (Additional file 2: Figure S3). To further verify the role of LKB1 in PIM-mediated AMPK phosphorylation, we used the CRISPR/Cas9-based genomic editing technique to knock out LKB1 from both PC3 and MCF7 cells (Additional file 2: Figure S1A). As demonstrated by DNA gel electrophoresis (Additional file 2: Figure S2) and Western blotting (Fig. 1b), there was no LKB1 expression in the knock-out cells. When AMPK phosphorylation levels were analysed, no significant differences were observed between wild-type and LKB1-deficient cells that had been treated with DMSO (Fig. 1b). By contrast, treatment with the PIM inhibitor DHPCC9 induced a profound increase in AMPK phosphorylation in wild-type, but not knock-out cells. Furthermore, transient expression of FLAG-tagged LKB1 in LKB1-deficient PC3 or MCF7 cells restored the response to DHPCC9 (Fig. 1c). Altogether, these data indicate that LKB1 is necessary for the PIM inhibition-induced increase in AMPK phosphorylation.

### AMPK phosphorylation levels are inversely correlated with PIM expression levels

**Fig. 2:**
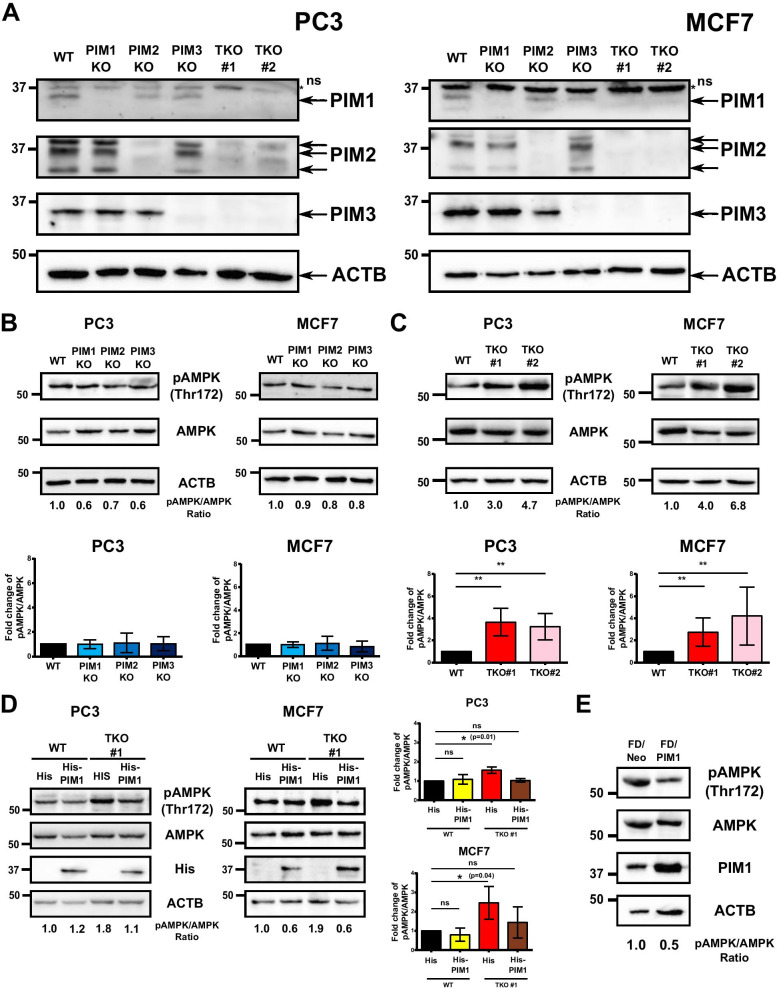
PIM expression levels inversely correlate with AMPK phosphorylation levels. PIM family members were knocked out in PC3 and MCF7 cells. **a** PIM expression levels in wild-type (WT) cells or their knock-out derivatives lacking individual (PIM1, PIM2, PIM3 KO) or all three (TKO) PIM kinases were examined by Western blotting. ACTB staining was used as a loading control. NS refers to non-specific staining observed with the PIM1 antibody. Phospho-AMPK (Thr172) versus AMPK levels were measured from WT cells in comparison to cells lacking individual **b** or all **c** PIM family members (average values ± SD, n = 3). **d** WT or TKO cells were transiently transfected with His or His-PIM1 plasmids, lysed 24 h later and subjected to Western blotting to determine relative AMPK phosphorylation levels (average values ± SD, n = 3). **e** Lysates of stably transfected FD/NEO and FD/PIM1 cell lines were subjected to Western blotting to determine relative AMPK phosphorylation levels

In order to analyse the respective contribution of the different PIM family members in regulating AMPK phosphorylation and activity, we used the CRISPR/Cas9 technique to generate both individual and combined PIM knock-out cells (Additional file 2: Figure S1B-D). As confirmed by DNA gel electrophoresis (Additional file 2: Figure S2) and Western blotting (Fig. 2a), single (KO) as well as triple (TKO) knock-out lines were successfully produced from both PC3 and MCF7 cells. Lack of any single PIM protein did not result in notable changes in AMPK phosphorylation in either cell line (Fig. 2b). By contrast, significantly elevated levels of phosphorylation were observed in the two independent PC3 and MCF7 TKO cell clones (Fig. 2c), while transient expression of His-tagged PIM1 in these cells reduced AMPK phosphorylation back to its basal level (Fig. 2d). Furthermore, FDCP1 myeloid cells stably overexpressing PIM1 (FD/PIM1) exhibited significantly lower levels of AMPK phosphorylation than the corresponding control cells (FD/NEO) (Fig. 2e). Taken together, our data indicate that either pharmacological inactivation or knock-out of all three PIM family members results in increased AMPK phosphorylation.

As AMPK is activated upon nutrient deprivation, we wanted to determine whether this response is affected by lack of PIM or LKB1 proteins. Therefore, we cultivated PC3-derived WT, PIM TKO and LKB1 KO cells in glucose-free medium supplemented with different concentrations of glucose and then analysed AMPK phosphorylation levels. As compared to WT cells, AMPK was more phosphorylated in PIM TKO cells and less phosphorylated in LKB1 KO cells (Additional file 2: Figure S4). However, glucose deprivation increased AMPK phosphorylation in all cells, suggesting that under such conditions, other kinases in addition to LKB1 regulate AMPK activity.

### LKB1 is a novel substrate for PIM kinases

**Fig. 3:**
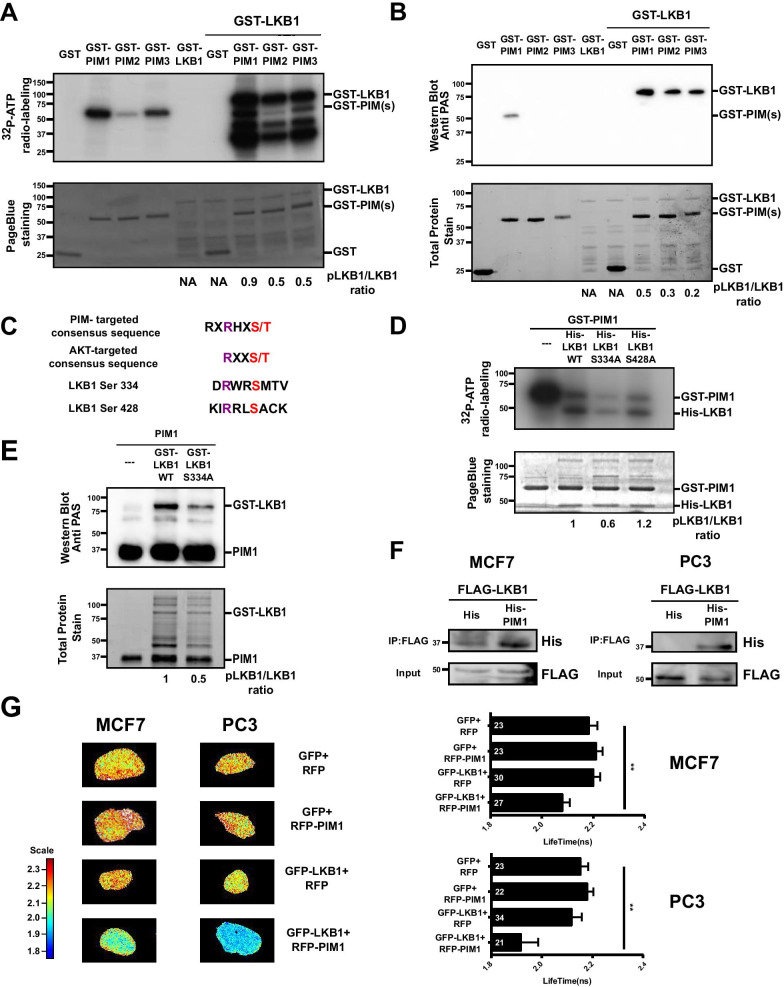
PIM kinases phosphorylate LKB1 in vitro and LKB1 interacts with PIM1 in cells. **a** Radioactive in vitro kinase assays were performed by incubating GST, GST-PIMs and/or GST-LKB1 in the presence of ^32^P-ATP. Phosphorylation signals were analysed by autoradiography (upper panel), while protein loading was visualised by Page Blue staining (lower panel). Shown is a representative image out of two repeated experiments. **b** Similar non-radioactive in vitro kinase assays were analysed by Western blotting with phospho-AKT substrate (PAS) antibody (upper panel), while protein loading was visualised by stain-free technology (lower panel). **c** Shown are the PIM kinase consensus phosphorylation motif, the PAS antibody recognition site, and the LKB1 sequences around residues Ser334 and Ser428. **d** Radioactive in vitro kinase assays (n = 2) were performed by incubating GST-PIM1 with His-tagged wild-type (WT) or mutant (S334A or S428A) LKB1. **e** Similar non-radioactive in vitro kinase assays (n = 2) were performed using GST-tagged PIM1 and LKB1 (WT or S334A) proteins. **f** MCF7 and PC3 cells were transiently transfected with FLAG-tagged LKB1 and His or His-tagged PIM1 plasmids. After 24 h, 10% of cell lysates were stained with FLAG antibody (input), while the rest was immunoprecipitated with FLAG M2 affinity gel and stained with His antibody (IP: FLAG). **g** MCF7 and PC3 cells were transiently transfected with GFP, GFP-tagged LKB1, RFP and/or RFP-tagged PIM1 plasmids. After 24 h, cells were fixed and analysed by fluorescence-lifetime imaging microscopy (FLIM). Shown are representative FLIM images as well as graphs (average value ± SD), where numbers of counted cells have been indicated

As LKB1 was indispensable for the PIM inhibitor-induced phosphorylation of AMPK in both PC3 and MCF7 cells, this raised the question of whether PIM kinases downregulate LKB1 activity by directly phosphorylating it. To address this question, we subjected GST-tagged PIM family members, LKB1 or their combinations to radioactive in vitro kinase assays. Visualisation of ^32^P-labeled phosphoproteins by autoradiography revealed that all three PIM kinases phosphorylate LKB1 in vitro, and that LKB1 does not undergo autophosphorylation (Fig. 3a). Thus, our data indicate that LKB1 indeed is a novel substrate targeted by all three PIM kinases. These data were further confirmed by a non-radioactive in vitro kinase assay (Fig. 3b), where phosphoproteins were visualised by Western blotting with the phospho-AKT substrate (PAS) antibody. This antibody recognises not only the AKT-targeted sequence RXXS/T, but also the PIM-targeted consensus sequence RXRHXS/T [39] (Fig. 3c).

For LKB1, multiple phosphorylation sites have been identified [40, 41]. However, only a few of them, including Ser334 and Ser428, resemble PIM target sites that can be recognised by the PAS antibody. To determine whether one or both of them are PIM target sites, we mutated them separately to alanine residues and subjected the mutant proteins to in vitro kinase assays. When His-tagged wild-type (WT) or mutant proteins were incubated in the presence of GST-PIM1, there was a 40% decrease in the intensity of the ^32^P-labeled signal for the S334A mutant as compared to the WT protein, while no significant changes were observed for the S428A mutant (Fig. 3d), indicating that Ser334 is a prominent PIM target site. This was confirmed by non-radioactive in vitro kinase assays followed by Western blotting with the PAS antibody (Fig. 3e). However, as the S334A mutation did not completely remove the residual signals in either assay, it remains possible that PIM kinases target also other sites in LKB1.

Having established PIM proteins as upstream kinases of LKB1, we examined their intracellular interactions. In co-immunoprecipitation assays, His-tagged PIM1 could be captured by FLAG-tagged LKB1 from both cell lines (Fig. 3f). In fluorescence-lifetime imaging microscopy (FLIM) analysis, significantly reduced GFP lifetimes were observed when GFP-tagged LKB1 and RFP-tagged PIM1 were co-expressed in either MCF7 or PC3 cells (Fig. 3g). Furthermore, in a proximity ligation assay (PLA) with anti-PIM1 and anti-FLAG antibodies, we observed significantly more colocalisation dots in PC3 cells between endogenously expressed PIM1 and ectopically expressed FLAG-tagged LKB1 than between PIM1 and FLAG (Additional file 2: Figure S5). All these data suggest that PIM1 and LKB1 physically interact with each other in cells.

### PIM kinases target Ser334 in LKB1 to regulate AMPK phosphorylation

**Fig. 4:**
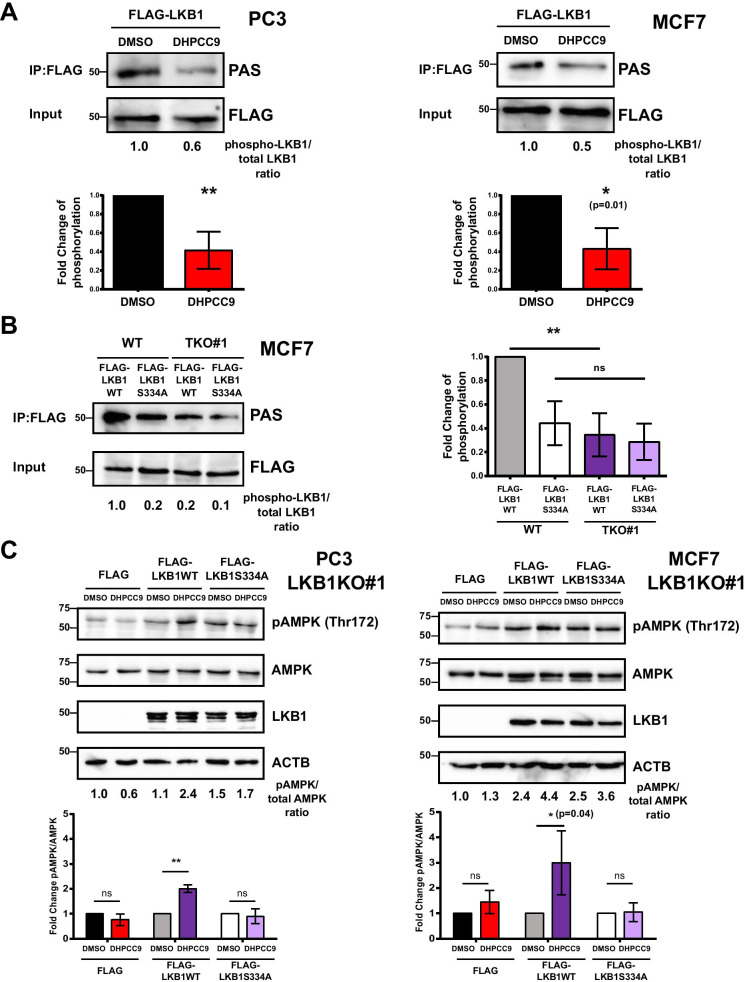
Ser334 is a PIM target site in LKB1 and is essential for increased AMPK phosphorylation in response to PIM inhibition. **a** PC3 and MCF7 cells were transiently transfected with the FLAG-LKB1 plasmid. After 24 h, cells were treated with either DMSO or 10 μM DHPCC9 for another 24 h. 10% of cell lysates were stained with FLAG antibody (Input), while the rest was immunoprecipitated with FLAG M2 affinity gel and stained with PAS antibody (IP:FLAG). Shown are representative images as well as graphs with relative differences in phosphorylation levels of LKB1 as compared to the DMSO-treated control samples (average values ± SD, n = 3). **b** MCF7 WT and TKO cells were transiently transfected with WT or S334A FLAG-LKB1 plasmids. After 48 h, relative phosphorylation levels of LKB1 were determined (average values ± SD, n = 3). **c** LKB1 KO derivatives of PC3 and MCF7 cells were transiently transfected with FLAG or FLAG-LKB1 (WT or S334A) plasmids. After 24 h, cells were treated with either DMSO or 10 μM DHPCC9 for another 24 h before Western blotting with pAMPK (Thr172), AMPK and LKB1 antibodies. Shown are representative images as well as graphs with relative phosphorylation levels of AMPK as compared to DMSO-treated control samples (average values ± SD, n = 3)

To verify that PIM kinases phosphorylate LKB1 in cells, we transiently expressed FLAG-tagged LKB1 in both PC3 and MCF7 cells. At 24 h after transfection, cells were treated with DMSO or 10 µM DHPCC9 for another 24 h, after which cells were lysed, FLAG-LKB1 proteins were pulled down with the FLAG antibody and their phosphorylation levels were analysed by Western blotting with the PAS antibody. As shown in Fig. 4a, the relative phosphorylation levels of LKB1 were significantly reduced by PIM inhibition in both types of cells. In addition to the pharmacological approach, we analysed LKB1 phosphorylation in WT and TKO MCF7 cells transiently expressing either FLAG-tagged LKB1 or the corresponding S334A mutant. In line with our data on PIM inhibition, LKB1 phosphorylation was dramatically decreased in TKO cells lacking all three PIM kinases (Fig. 4b). Notably, there was no significant difference between the phosphorylation level of the S334A mutant in WT and TKO cells, suggesting that Ser334 is a prominent PIM target site in LKB1.

To explore the impact of Ser334 phosphorylation of LKB1 on AMPK phosphorylation, FLAG-tagged LKB1 or the corresponding S334A mutant were transiently expressed in LKB1 KO derivatives of PC3 and MCF7 cells, and the cells were treated with either DMSO or 10 µM DHPCC9. As expected, DHPCC9 treatment did not trigger any considerable increase in AMPK phosphorylation in either type of FLAG-transfected cells lacking LKB1 (Fig. 4c). By contrast, reintroduction of WT LKB1, but not the phosphorylation-deficient S334A mutant restored the response of cells to DHPCC-9, resulting in a significant increase in AMPK phosphorylation. These data suggest that phosphorylation of LKB1 at Ser334 is involved in the regulation of AMPK phosphorylation by PIM kinases and their inhibitors.

AKT has been reported to phosphorylate LKB1 at Ser334, resulting in its nuclear sequestration by the 14–3–3 protein [42]. To determine whether PIM-dependent phosphorylation of this site has similar consequences in PC3 or MCF7 cells, we fractionated LKB1KO cells transiently expressing FLAG-tagged WT LKB1 or the phosphodeficient S334A mutant. According to our analyses, both WT and mutant proteins were mainly localised in the nuclear fractions of both PC3 and MCF7 derivatives (Additional file 2: Figure S6A). By contrast, the endogenous LKB1 in parental cells was mostly localised in the cytosolic fractions, and this was not influenced by pharmacological PIM inhibition (Additional file 2: Figure S6B) or by knocking out of all three PIM kinase members (Additional file 2: Figure S6C). However, the level of AMPK phosphorylation in the cytoplasmic fraction was increased in both cases. To confirm that there is no compensatory activation of AKT in the PIM TKO cells, we analysed AKT Ser473 phosphorylation levels from them, but did not observe any major changes as compared to WT cells (Additional file 2: Figure S6D).

### Combined knock-out of LKB1 and PIM kinases impairs cell proliferation and tumor growth

**Fig. 5:**
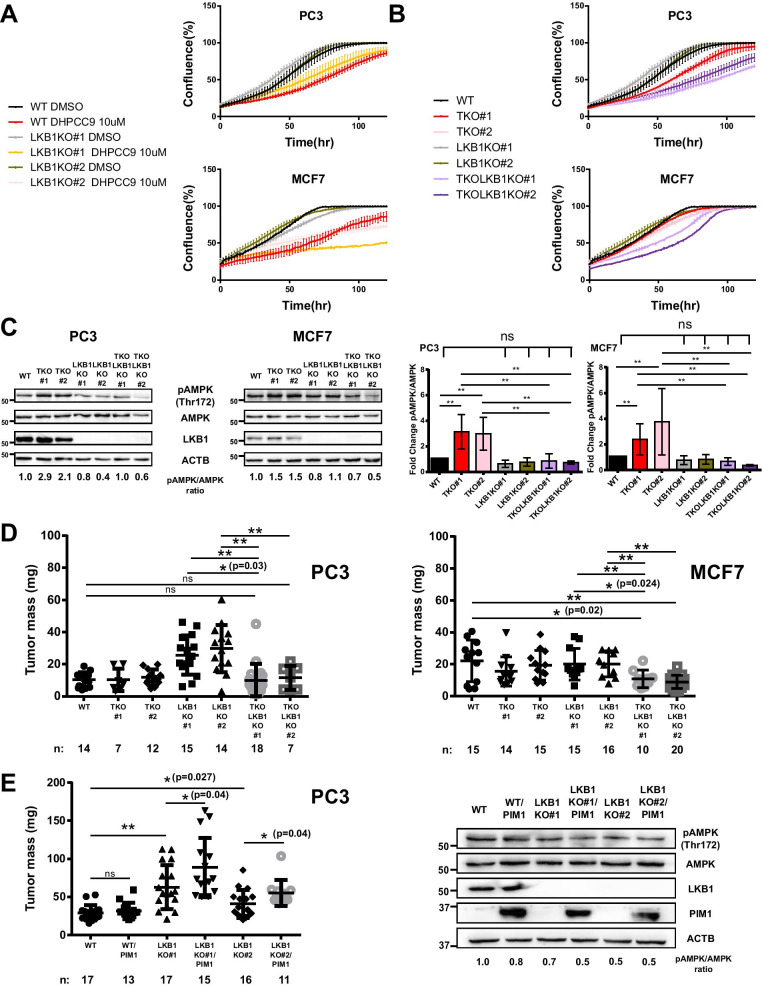
Lack of both LKB1 and PIM kinases impairs cell proliferation and tumor growth**. a** WT PC3 or MCF7 cells or their LKB1-deficient KO derivatives were grown overnight on 96-well plates, after which they were treated with DMSO or 10 μM DHPCC9, and their proliferation was followed for 5 days using the IncuCyte live cell imaging system. Shown are average percentages of confluence at indicated time-points (± SD of a representative experiment, n = 3). **b** Proliferation assays were performed with WT PC3 or MCF7 cells or their KO or TKO derivatives lacking LKB1 and/or all PIM kinases, respectively (n = 3). **c** Phospho-AMPK (Thr172) versus AMPK levels were measured from WT cells in comparison to cells lacking all PIM family members or LKB1 or both PIM and LKB1 in PC3 and MCF7 cells (average values ± SD, n = 3). **d** WT PC3 and MCF7 cells or their KO and/or TKO derivatives were grown for 7 days on the chorioallantoic membranes (CAM) of chick embryos. Shown are scatter plots of tumor mass at the end of the experiment. Numbers of the samples are listed at the bottom of the graphs. **e** PC3 cells and their LKB1KO derivatives were transiently transfected with His or His-PIM1 plasmids for 48 h before being grown for 7 days on CAM. Parts of the xenograft samples were subjected to Western blotting to examine PIM1 and LKB1 expression levels. Shown are scatter plots of tumor mass at the end of the experiment. Numbers of the samples are listed at the bottom of the graphs

We next performed an in silico analysis of mRNA expression levels in patient-derived samples and observed that in prostate carcinomas and certain breast carcinomas, *PIM* expression was elevated and *LKB1/STK11* expression was reduced (Additional file 2: Figure S7). However, in the breast medullary carcinoma dataset, both *PIM* and *LKB1* expression levels were highly upregulated. As LKB1 expression and LKB1-dependent AMPK activation are often associated with cell growth suppression [43], this prompted us to evaluate the proliferation rates for WT PC3 or MCF7 cells or their LKB1-deficient derivatives in response to treatment with DMSO or 10 µM DHPCC9. Proliferation was followed for 5 days by measuring cell confluence with the IncuCyte live cell imaging system, where the two independent LKB1 KO clones behaved similarly to the WT cells (Fig. 5a). All the DMSO-treated cells proliferated well with sigmoidal growth curves, while the DHPCC9 treatment retarded cell growth.

The proliferation rates of TKO cells lacking all PIM kinases were reduced (Fig. 5b), which was in line with what we observed following the DHPCC9 treatment of WT cells. Surprisingly, cells lacking both LKB1 and PIM kinases (TKOLKB1 KO) grew even slower than TKO clones. As shown in Fig. 5c, increased AMPK phosphorylation levels in TKO clones correlated well with their slower proliferation rates as compared to their WT counterparts. On the other hand, due to the absence of LKB1, the phosphorylation levels of AMPK in TKOLKB1 KO clones were lower than in TKO clones, yet the proliferation rates of TKOLKB1 KO clones were slower. These data suggest that under the conditions of PIM inhibition, changes in AMPK phosphorylation levels are not directly connected to the proliferation properties of LKB1 KO cells.

We next employed the chick embryo chorioallantoic membrane (CAM) xenograft model [37] to investigate the in vivo behaviour of the knock-out cells lacking both PIM and LKB1. WT MCF7 and PC3 cells or their KO and/or TKO derivatives were implanted onto the CAM of eggs on day 7 of incubation to allow the development of tumors. On day 14, tumors were excised from the CAM and weighed. Interestingly, there was a significant increase in the mass of tumors derived from LKB1 KO clones in PC3 but not MCF7 cells (Fig. 5d), but there was no significant difference between WT and TKO clones in either cell type. However, the mass of TKOLKB1KO MCF7 tumor cells was significantly lower than that of WT or LKB1KO samples. Intriguingly, while loss of LKB1 alone in PC3 cells increased tumor load, this effect was abolished, when combined with the loss of PIM kinases. Conversely, transient over-expression of PIM1 triggered further increases in tumor mass in two independent PC3 LKB1KO clone xenografts but not in WT samples (Fig. 5e). These data indicate that LKB1 and PIM kinases cooperate in the regulation of tumorigenic growth.

## Discussion

To our knowledge, this is the first report combining both pharmacological and CRISPR/Cas9-based genomic editing approaches to show that inhibition of the expression or activity of all three PIM kinases activates AMPK in cancer cells via LKB1-dependent phosphorylation at Thr172. Notably, knocking out of any particular PIM family member is not sufficient to trigger AMPK activation, reflecting the previously observed functional redundancy of PIM kinases and the fact that all three PIM kinases are capable of phosphorylating LKB1 and thereby inhibiting its ability to phosphorylate AMPK. Besides demonstrating that PIM kinases are upstream regulators of LKB1, we have also identified Ser334 as the major, although possibly not the sole PIM target site in LKB1.

In MDA-MB-231 breast cancer cells, phosphorylation of LKB1 at Ser334 by AKT has been reported to block the tumor suppressor activity of overexpressed LKB1 via nuclear sequestration by the 14–3–3 protein [42]. However, there may be cell type-specific differences in the subcellular localisation of LKB1. While endogenously expressed LKB1 protein is exclusively localised in the nucleus of non-transformed IMR90 fibroblasts, it is predominantly located in the plasma membrane of polarised epithelial MDCK cells [43]. According to our fractionation data, overexpressed LKB1 and its S334A phosphodeficient mutant derivative are both mostly found in the nuclear fractions of PC3 and MCF7 LKB1KO cells, while the endogenously expressed LKB1 protein of the parental cells resides in the cytoplasm, irrespective of whether PIM expression or activity is inhibited. These discrepancies in the cellular compartmentalisation between endogenous and ectopically expressed proteins warrants the usage of knock-in mutant cell lines to properly examine the physiological consequences of LKB1 phosphorylation.

It is not surprising that both PIM and AKT kinases target LKB1, as they also share several other substrates [2, 3]. For example, both PIM and AKT protect cells from apoptosis by phosphorylating the pro-apoptotic BAD protein, albeit at different but proximate sites [44, 45]. In addition, both PIM and AKT promote mTOR- and cap-dependent protein synthesis by phosphorylating the AKT1S1 and EIF4EBP1 translational inhibitors [2, 3]. However, these kinases also have cell type-specific non-redundant roles, as we did not detect any compensatory increase in AKT activity in PC3 or MCF7 PIM TKO cells.

In terms of cell proliferation and tumor growth, the tumor-suppressive effects of LKB1 could be readily seen in the chick embryo CAM xenograft experiments, but not in the two-dimensional cell proliferation assays. Similar discrepancies with respect to the effects of LKB1 between in vitro and in vivo models have also recently been demonstrated [46]. Co-deletion of PTEN and LKB1 from prostate cancer cells results in aggressive tumors and lung metastases, while deletion of LKB1 alone has no such effect. This finding is in line with our CAM data, in which knocking out LKB1 elicited a robust increase in tumor mass in PTEN-deficient PC3 cells, but not in PTEN-expressing MCF7 cells. In PC3 cells, the resulting oncogenic insult could be either suppressed by knocking out all three PIM kinase members or exacerbated by upregulating PIM1 expression, highlighting the integral role of PIM kinases in supporting tumor growth in this setting. Notably, the combined PIM and LKB1 knock-out slowed the rate of cell proliferation and tumor growth as compared to the LKB1 knock-out alone, but without considerable changes in AMPK phosphorylation levels. Even though a decrease in AMPK activity is conventionally associated with the enhanced growth of tumors lacking LKB1, this idea has recently been challenged by findings in K-Ras-driven models of non-small-cell lung carcinoma which indicated that loss of LKB1 and AMPK suppresses tumorigenesis [47]. In addition, emerging data have revealed that loss of salt-inducible kinases (SIKs), which are less-well studied LKB1 downstream targets, accounts for a significant proportion of the transcriptional changes and histological features of LKB1-deficient tumors [48, 49]. Further studies are therefore needed to determine whether PIM kinases share signaling pathways with SIKs in affecting the growth of LKB1-deficient tumors as well as whether PIM inhibition can suppress the aggressive metastatic behaviour observed in tumors lacking both PTEN and LKB1.

## Conclusions

Catabolic events invoked by the LKB1/AMPK signalling pathway are expected to antagonise the oncogenicity of PIM kinases. Our novel finding that PIM kinases act as upstream regulators of LKB1 uncovers a molecular pathway that allows the tumor-suppressive function of LKB1 and the oncogenic functions of PIM kinases to be tightly and precisely controlled. Inactivation of both PIM kinases and LKB1 results in a significant decrease in cell proliferation in vitro and tumor growth in vivo, suggesting that PIM-targeted pharmaceutical interventions could be exploited to suppress the growth of LKB1-deficient tumors.

## Supplementary Information

**Additional file 1: Table S1.** Primers for mutagenesis. **Table S2**. CRISPR sgRNA sequences. **Table S3**. Primers for verification of CRISPR knock-out clones. **Table S4**. Antibodies.

**Additional file 2: Figure S1** Schematic diagram for the strategies of CRISPR/Cas9 design and verification. **Figure S2**. DNA gel electrophoresis results for wild-type and knock-out clones. **Figure S3**. LKB1 is needed for PIM-dependent regulation of AMPK phosphorylation. **Figure S4**. Glucose deprivation increases AMPK phosphorylation in an LKB1-independent fashion. **Figure S5**. Proximity ligation assay (PLA) to demonstrate the physical interactions between PIM1 and LKB1. **Figure S6**. Analyses of LKB1 subcellular localisation and AKT phosphorylation levels. **Figure S7**. Expression of PIM family members and LKB1(STK11) in distinct types of breast or prostate cancer.

## Data Availability

PhosphoSitePlus® database (https://phosphosite.org) and IST Online™ database (https://ist.medisapiens.com) were used for in silico analyses.

## Abbreviations

AMPK: AMP-activated protein kinase
CAM: chorioallantoic membrane
FLIM: fluorescence-lifetime imaging microscopy
PLA: proximity ligation assay
KO: knock-out
LKB1: liver kinase B1
PAS: phosphorylated AKT substrate
SA: serine (S) to alanine (A) phosphomutant
TKO: triple knock-out
WT: wild-type
