# Supplementary material for: PIM kinases inhibit AMPK activation and promote tumorigenicity by phosphorylating LKB1
Source: Cell Commun Signal. 2021 Jun 30;19:68. doi: 10.1186/s12964-021-00749-4 (PMC8247201; doi:10.1186/s12964-021-00749-4)
Supplement: Supplementary file 2 — Additional file 1: Table S1. Primers for mutagenesis. Table S2. CRISPR sgRNA sequences. Table S3. Primers for verification of CRISPR knock-out clones. Table S4. Antibodies. [file 12964_2021_749_MOESM2_ESM.pdf]

Table S1. Primers for mutagenesis

Target	Primer Type	Sequence
LKB1S334A	Forward	CACCACAGTCATGGCGCGCCACCGGTCC
LKB1S334A	Backward	GGACCGGTGGCGCGCCATGACTGTGGTG
LKB1S428A	Forward	GCTTGCAGGCCGCCAGCCGGCGG
LKB1S428A	Backward	CCGCCGGCTGGCGGCCTGCAAGC
LKB1Sequencing Primer	Forward	GAAGGGGACAACATCTACAAGT

Table S2. CRISPR sgRNA sequences

Target Location	Sequence
PIM1 Exon2	CCGGCAAGTTGTCGGAGACG
PIM1 Exon6	TCGAAGGTTGGCCTATCTGA
PIM2 Exon2	TTCGAGGCCGAGTATCGACT
PIM2 Exon6	GGCCAGGCACCGGCGGATTA
PIM3 Exon5	GGGCGTGCTTCTCTACGATA
PIM3 Exon6	GCCGTCGCTGGATCAGATTG
LKB1 Exon3	CACCCTCAAAATCTCCGACC
LKB1 Exon7	CATGCTGCGCCACCGGTCCT

Table S3. Primers for verification of CRIPSR knock-out clones

Target	Primer Type	Sequence
PIM1 Exon2(P1E2)	Forward	ATGCTCTTGTCCAAAATCAACTCG
PIM1 Exon6(P1E6)	Forward	ATGCTTGGCCTCCCTGG
PIM1 Exon6(P1E6)	Backward	GAGAGTCACTCTTCCCCACTGTT
PIM2 Exon2(P2E2)	Forward	CAAGCCTCTACAGGGGC
PIM2 Exon2(P2E2)	Backward	TCCTCTACACACTCTGCAGG
PIM2 Exon6(P2E6)	Backward	GTAAAACCAAGTCAACAAATGTCC
PIM3	Forward	GGTGATGACGAGCAGGATTT
PIM3	Backward	TTTGGACAGACAGAGCTTGAG
LKB1	Forward	CAAAGGGGACCCCTGTGAG
LKB1	Backward	GTCCGGCAGGTGTCGTC

Table S4. Antibodies

Protein or tag	Company	Product Code	Dilution
PIM1	Santa Cruz	12H8	1:500
PIM2	Cell Signaling Technology	#4730	1:1000
PIM3	Cell Signaling Technology	#4165	1:1000
PIM1 (PLA assay)	Merck	MABC553	1:500
FLAG-tag (PLA assay)	Sigma	F7425	1:500
Beta-actin	Cell Signaling Technology	#3700	1:5000
Phospho-AMPKα (Thr172)	Cell Signaling Technology	#2325	1:1000
AMPKα	Cell Signaling Technology	#2793	1:1000
LKB1	Cell Signaling Technology	#3047	1:1000
His-tag	Cell Signaling Technology	#12698	1:1000
FLAG-tag	Sigma	F1804	1:1000
Phospho-Akt Substrate (RXXS/T)	Cell Signaling Technology	#9614	1:1000
β-Tubulin	Cell Signaling Technology	#86298	1:5000
Lamin A/C	Cell Signaling Technology	#4777	1:5000
Phospho-Akt (Ser473)	Cell Signaling Technology	#4060	1:2000
Akt	Cell Signaling Technology	#9272	1:2000
