# Supplementary material for: PIM kinases inhibit AMPK activation and promote tumorigenicity by phosphorylating LKB1
Source: Cell Commun Signal. 2021 Jun 30;19:68. doi: 10.1186/s12964-021-00749-4 (PMC8247201; doi:10.1186/s12964-021-00749-4)
Supplement: Supplementary file 3 — Additional file 2: Figure S1 Schematic diagram for the strategies of CRISPR/Cas9 design and verification. Figure S2. DNA gel electrophoresis results for wild-type and knock-out clones. Figure S3. LKB1 is needed for PIM-dependent regulation of AMPK phosphorylation. Figure S4. Glucose deprivation increases AMPK phosphorylation in an LKB1-independent fashion. Figure S5. Proximity ligation assay (PLA) to demonstrate the physical interactions between PIM1 and LKB1. Figure S6. Analyses of LKB1 subcellular localisation and AKT phosphorylation levels. Figure S7. Expression of PIM family members and LKB1(STK11) in distinct types of breast or prostate cancer. [file 12964_2021_749_MOESM3_ESM.pdf]

Figure S1

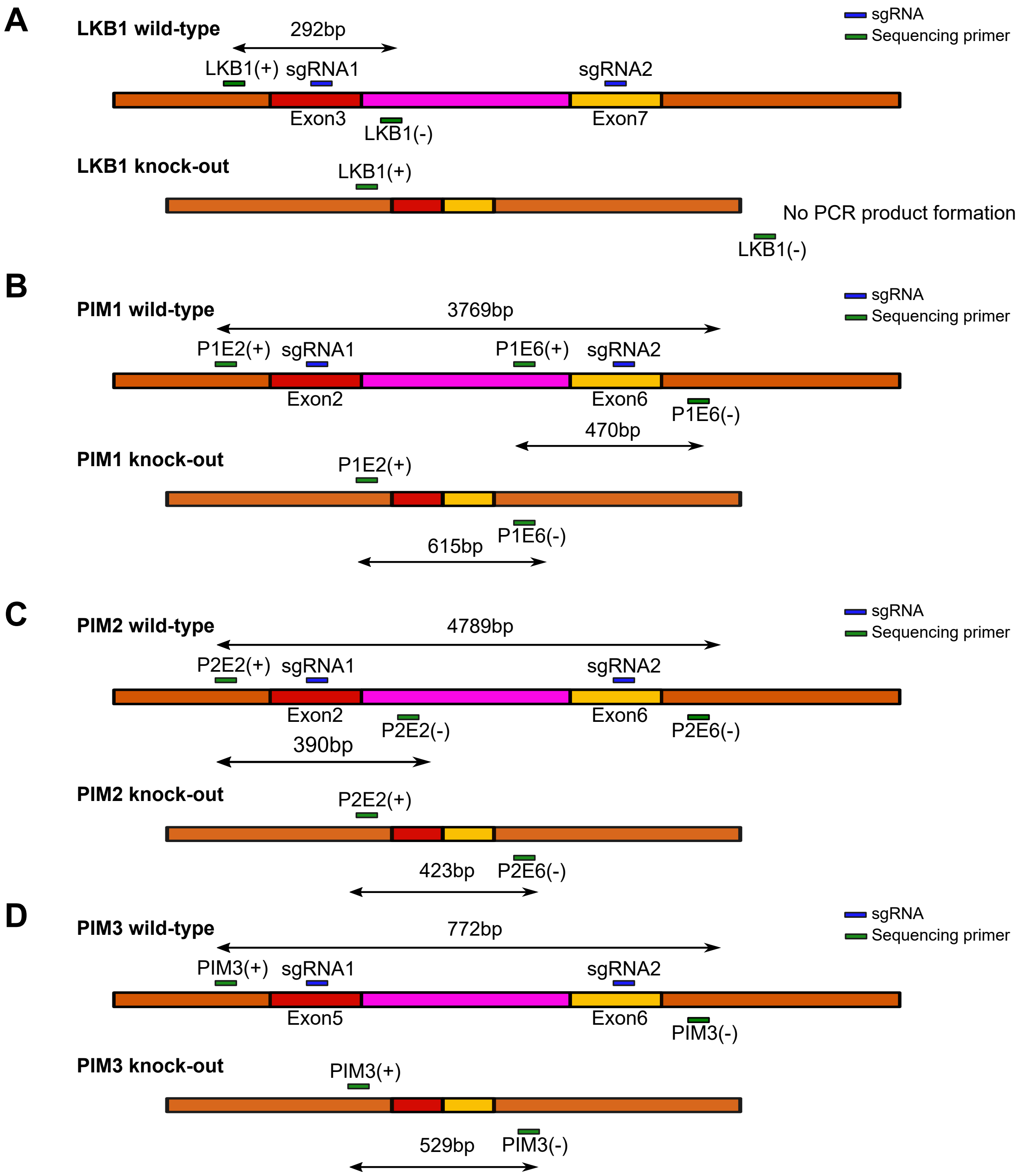

Figure S1. Schematic diagram for the strategies of CRISPR/Cas9 design and verification. To knock out desired genes, two sgRNAs (coloured in blue) were chosen that targeted genomic DNA within exon 2 and 6 of PIM1 (**A**), exon 2 and 6 of PIM2 (**B**), exon 5 and 6 of PIM3 (**C**), or exon 3 and 7 of LKB1(**D**). Sequencing primers (coloured in green) were chosen for nearby sites to be able to verify the wild-type (WT) and the corresponding knock-out (KO) clones.

Figure S2

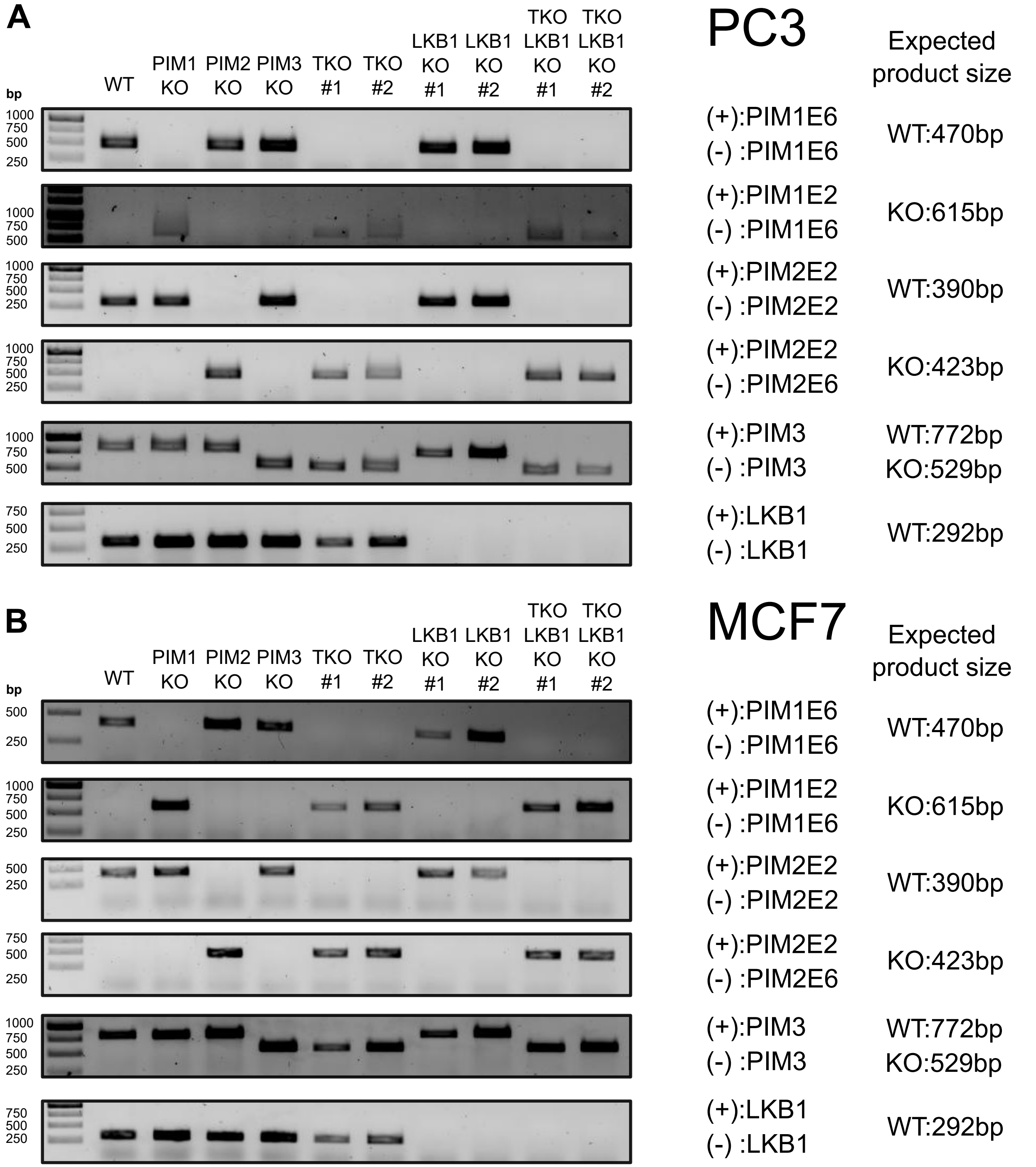

Figure S2. DNA gel electrophoresis results for wild-type and knock-out clones. Pairs of indicated sequencing primers were used to amplify genomic DNA from PC3 (**A**) and MCF7 (**B**) wild-type (WT) cells, their knock-out derivatives lacking individual (PIM1, PIM2, PIM3 KO) or all three PIM kinases (TKO), LKB1 (LKB1KO) or LKB1 plus all three PIM kinases (TKOLKB1KO). Amplicons with expected product sizes are listed. Products longer than 2 kb were not amplified, as PCR extension time was set to 1 min (2kb/min) in each amplification cycle.

Figure S3

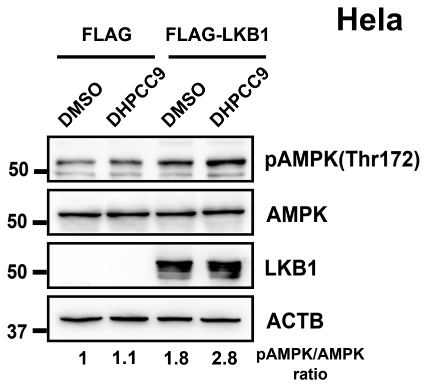

Figure S3. LKB1 is needed for PIM-dependent regulation of AMPK phosphorylation. Hela cells transiently expressing FLAG or FLAG-tagged LKB1 were treated for 24h with DMSO or 10µM DHPCC9, and subjected to Western blotting with antibodies against phospho-AMPK (Thr172), AMPK or LKB1. ACTB staining was used as a loading control. Shown below the graph are the relative levels of phosphorylated versus total AMPK that were quantitated in comparison to DMSO-treated control samples.

Figure S4

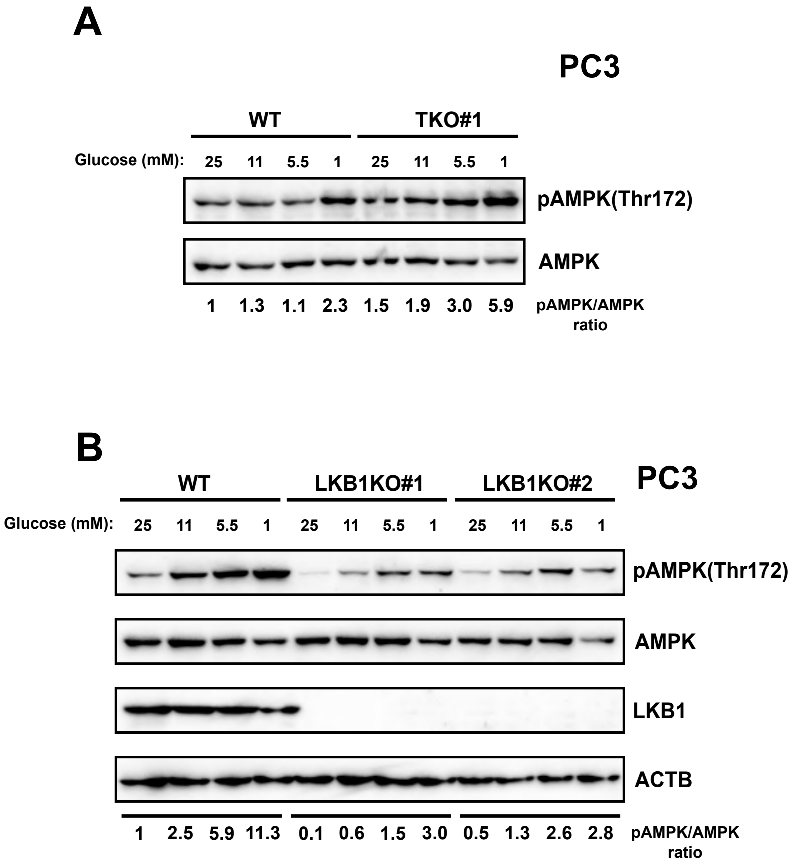

Figure S4. Glucose deprivation increases AMPK phosphorylation also in an LKB1-independent fashion. **A**) PC3 WT and TKO cells were incubated with indicated glucose concentrations for 24h, and subjected to Western blotting. **B**) PC3 WT and LKB1KO cells were incubated with indicated glucose concentrations for 48h, and subjected to Western blotting. ACTB staining was used as a loading control. Shown below the graphs are the relative levels of phosphorylated versus total AMPK that were quantitated in comparison to control samples of WT cells grown in the presence of 25 mM glucose.

Figure S5

A

PC3

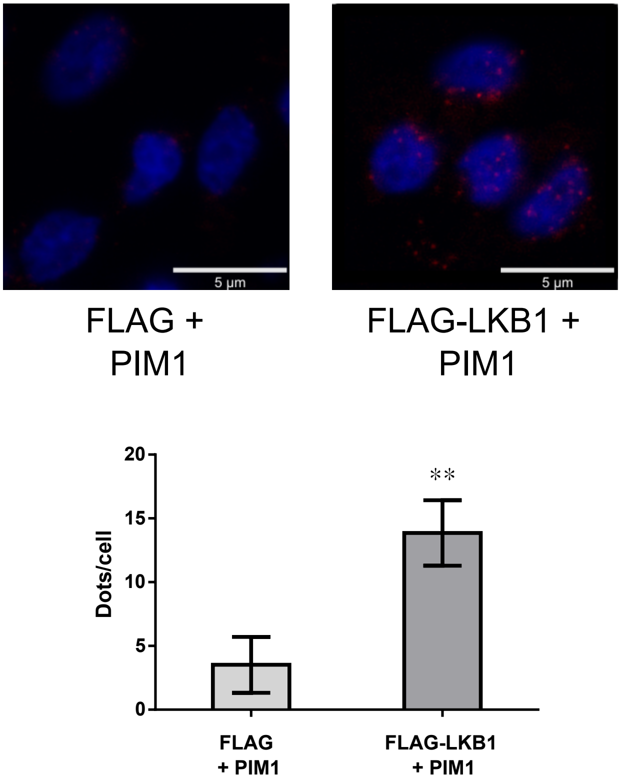

Figure S5. Proximity ligation assay (PLA) to demonstrate the physical interactions between PIM1 and LKB1. PC3 cells transiently expressing FLAG or FLAG-tagged LKB1 plasmids were imaged 24h after transfection. Shown are representative images and quantification from the PLA assays with anti-PIM1 and anti-FLAG antibodies.

Figure S6

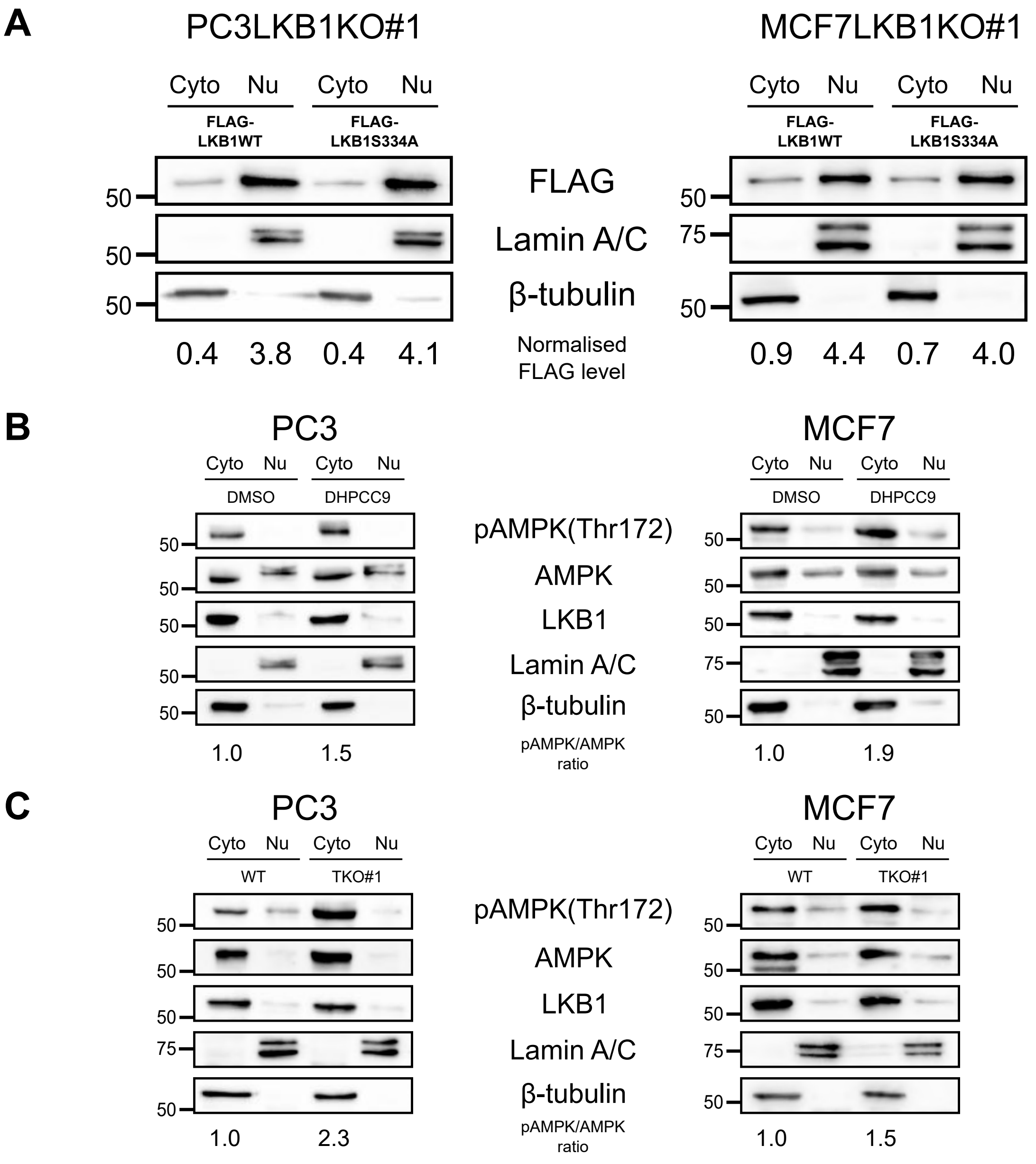

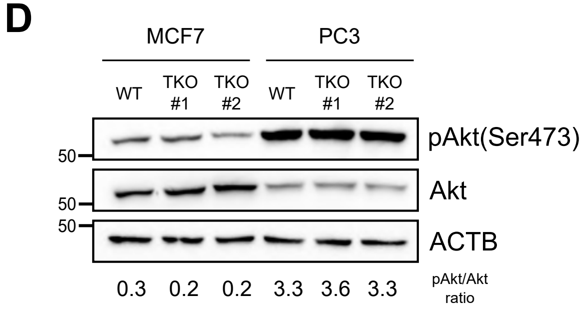

Figure S6. Analyses of LKB1 subcellular localisation and AKT phosphorylation levels. **A**) FLAG-tagged WT LKB1 and its Ser334A phosphodeficient mutant were transiently overexpressed in LKB1KO derivatives of PC3 or MCF7 cells and fractionated after lysis. β-Tubulin and Lamin A/C were used as markers for cytosolic (Cyto) and nuclear (Nu) fraction, respectively. The effects of **B**) PIM inhibition by DHPCC9 or **C**) PIM triple knock-out (TKO) on localization of endogenously expressed LKB1 and on AMPK phosphorylation levels were analysed from fractionated samples. **D**) Phosphorylation levels of AKT at Ser 473 and overall AKT expression levels were examined in both PC3 and MCF7 WT and TKO clones.

Figure S7

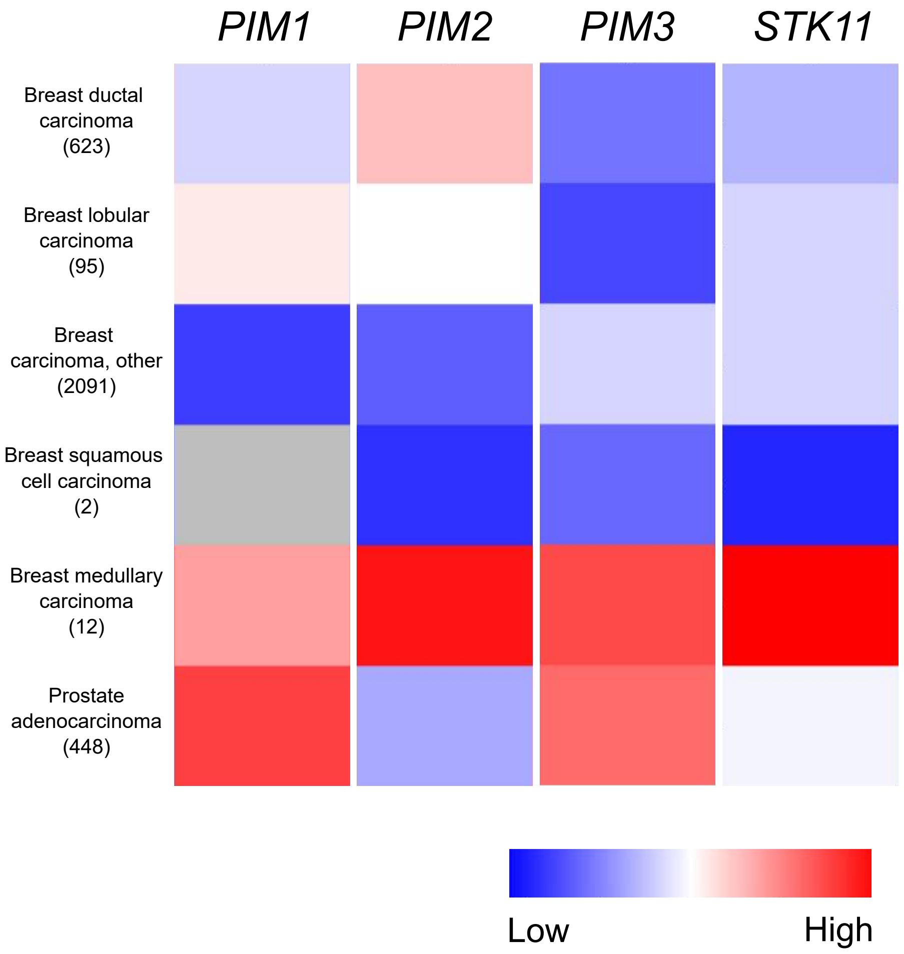

Figure S7. Expression of *PIM* family members and *LKB1(STK11)* in distinct types of breast or prostate cancer. Gene expression heatmap for *PIM1, PIM2, PIM3* and *LKB1(STK11)* in breast and prostate cancer subtypes were generated from IST Online™ database (ist.medisapiens.com). The colour in the heatmap refers to the mean mRNA expression values. Numbers in the brackets stand for the number of patient-derived samples available for analysis.
